# Long-COVID: assessment of circulating markers suggests no cerebral neuronal damage, neuroinflammation or systemic inflammation–a controlled study

**DOI:** 10.1038/s41598-026-40142-0

**Published:** 2026-03-03

**Authors:** Roald Omdal, Ole Bernt Lenning, Grete Jonsson, Jan Terje Kvaløy, Guglielmo Di Molfetta, Kubra Tan, Andrea L. Benedet, Nicholas J. Ashton, Geir Sverre Braut, Henrik Zetterberg, Tore Grimstad

**Affiliations:** 1 Research Department, Stavanger University Hospital, Stavanger, Norway; 2 Department of Clinical Science, University of Bergen, Bergen, Norway; 3 Department of Medical Biochemistry, Stavanger University Hospital, Stavanger, Norway; 4 Department of Mathematics and Physics, University of Stavanger, Stavanger, Norway; 5 Department of Psychiatry and Neurochemistry, Institute of Neuroscience and Physiology, The Sahlgrenska Academy at the University of Gothenburg, Mölndal, Sweden; 6 Banner Alzheimer’s Institute and University of Arizona, Phoenix, AZ USA; 7 Banner Sun Health Research Institute, Sun City, AZ 85351 USA; 8 The Western Norway, University of Applied Sciences, Sogndal, Norway; 9 Clinical Neurochemistry Laboratory, Sahlgrenska University Hospital, Mölndal, Sweden; 10 Department of Neurodegenerative Disease, UCL Institute of Neurology, Queen Square, London, UK; 11 UK Dementia Research Institute at UCL, London, UK; 12 Hong Kong Center for Neurodegenerative Diseases, InnoHK, Hong Kong, China; 13 Wisconsin Alzheimer’s Disease Research Center, School of Medicine and Public Health, University of Wisconsin, University of Wisconsin-Madison, Madison, WI 53792 USA; 14 Centre for Brain Research, Indian Institute of Science, Bangalore, India; 15 Department of Internal Medicine, Stavanger University Hospital, Stavanger, Norway

**Keywords:** Long-COVID, Neuroinflammation, Innate immunity, Sickness behavior, Biomarkers, Diseases, Immunology, Medical research, Neurology, Neuroscience

## Abstract

Long-COVID remains incompletely understood, particularly regarding the roles of peripheral systemic inflammation and neuroinflammation. The persistence and extent of these processes remain debated. We conducted a single-center, age- and sex-matched case–control study at Stavanger University Hospital, Norway, recruiting participants from the general population. Forty-eight long-COVID patients and 48 recovered controls were included at a median of 69 weeks post-SARS-CoV-2 infection. Exclusion criteria included autoimmune or chronic inflammatory diseases, cancer, and other conditions affecting fatigue. Plasma levels of neurofilament light (NfL), glial fibrillary acidic protein (GFAP), triggering receptor expressed on myeloid cells 2 (TREM2), C-reactive protein (CRP), tumor necrosis factor-α (TNF-α), and interleukin-6 (IL-6) were measured using ultrasensitive NULISA™ technology. CRP, TNF-α, and IL-6 were additionally assessed by a standard hospital laboratory method (CRP) and MSD S-Plex chemiluminescence immunoassay (TNF-α and IL-6 MSD). No significant differences in NfL or GFAP were observed between groups, suggesting no ongoing neuronal injury or neuroinflammation. Routine immunoassays showed no differences for inflammatory markers. In unadjusted analyses using ultrasensitive assays, long-COVID patients showed nominally elevated levels of CRP (*p* = 0.04), TNF-α (*p*  = 0.01), IL-6 (*p* = 0.02), and TREM2 (*p* =  0.02). However, these differences did not survive correction for multiple comparisons (all false discovery rate-adjusted *p* > 0.05). The absence of neuroinflammation markers is consistent with the hypothesis that persistent long-COVID symptoms are unlikely due to ongoing neuronal injury or central nervous system inflammation. Alternatively, persisting long-COVID symptoms may reflect a chronic, extremely low-level immune activation, that contributes to fatigue, pain, and other sickness phenomena through mechanisms such as pro-inflammatory signaling in the brain, or epigenetic mechanisms underlying the sickness behavior response. These findings should be considered preliminary and warrant validation in larger, longitudinal cohorts.

## Introduction

Long-COVID (LC) is an emerging global health challenge, with its prevalence increasing upon repeated exposures to SARS-CoV-2, irrespective of the severity of the initial infection^1^. Between 2020 and 2024, the estimated global prevalence of LC increased from 60 to 400 million, with a prevalence of approximately 6–7% in adults and 1% in children. Long-term observational studies remain limited. Some studies with observation periods of up to 24 months suggest that symptoms persist largely unchanged over time^2,3^. In contrast, others report a more favourable trajectory, with symptoms becoming less severe as time passes^4^. Additional data and extended follow-up studies are needed to clarify these trends further.

While acute COVID-19 is widely recognized as a multi-organ disorder with direct tissue involvement affecting the immune, cardiovascular, and/or central nervous systems, and potentially involving organs such as the gastrointestinal tract, kidneys, and lungs^1^, the mechanisms driving the chronic phase remain debated l^5^. Some hypotheses emphasize persistent organ damage and reactivated viral reservoirs, while others propose that LC may resemble other post-infectious syndromes where symptoms persist without clear evidence of ongoing structural organ damage.

Numerous studies have identified various biomarker abnormalities in patients, though findings are not always consistent. These abnormalities include elevated CRP levels, increased proinflammatory cytokines and chemokines, activation of the adaptive immune system, and laboratory evidence of target organ involvement, such as neuronal cell damage in the brain and thromboembolism^6^. Notably, many of these studies were conducted relatively early during LC, within a few months after the SARS-CoV-2 infection. This timing raises the possibility that the findings may reflect ongoing viral presence or organ damage still in the process of healing.

Early studies reported a wide range of symptoms and findings. Over time, however, this picture may have shifted, with fatigue and subjective cognitive impairment—often described by patients as “brain fog”—emerging as the main features of LC^3,4,7^.

These observations align with findings of post-viral illnesses following infections such as poliovirus, CMV, and EBV where symptoms often become chronic while peripheral inflammatory markers typically normalize or remain low after the acute phase^8–12^. If LC is indeed a form of post-viral fatigue syndrome, it could be hypothesized that it would share similarities with such conditions, which typically exhibit minimal or no evidence of inflammatory markers, immune system activation, or ongoing organ damage.

This hypothesis also aligns with findings from our 65-week post-infection study, which compared LC patients to individuals who recovered without LC. We observed no significant differences in systemic inflammation or mast cell activation^13^. Similarly, other studies conducted six months or more after SARS-CoV-2 infection suggest minimal residual inflammation or neurological damage in LC patients^14–16^.

To evaluate whether ongoing neuroinflammation or other pathological processes impacting cerebral structures, neuronal integrity, function, or homeostasis persist in LC patients over time, we analysed blood levels of neurofilament light (NfL) and glial fibrillary acidic protein (GFAP) at a median of 69 weeks post-SARS-CoV-2 infection. NfL and GFAP are well-established and validated biomarkers of cerebral “health”^17^. NfL reflects neuronal degradation, damage or inflammation, while GFAP serves as a marker of astroglial-driven neuroinflammation. Previous studies have reported elevated levels of these biomarkers in LC patients, suggesting ongoing cerebral involvement^18,19^.

Additionally, we measured plasma levels of triggering receptor expressed on myeloid cells 2 (TREM2), a transmembrane receptor primarily expressed on microglia in the brain and on peripheral myeloid cells including monocytes and macrophages^20^. TREM2 is upregulated during immune responses to viral and non-viral pathogens, and elevated levels have been reported in acute and severe COVID-19, where they correlate with disease severity^21^. Soluble TREM2 (sTREM2) in plasma reflects myeloid cell activation and has emerged as a biomarker of neuroinflammation and microglial activity ^22^. Plasma TREM2 reflects total myeloid cell activation (both central and peripheral) and thus cannot be definitively attributed to neuroinflammation alone. However, the measurement of TREM2 allows us to assess whether the myeloid cell activation characteristic of acute infection persists in the chronic phase of LC.

To assess potential ongoing peripheral systemic inflammation, we also measured classical inflammatory markers in blood, including C-reactive protein (CRP), tumor necrosis factor alpha (TNF-α), interleukin (IL)-1β, and IL-6 using both NULISA and more conventional immunoassays.

## Methods

### Study design and patient recruitment

This single-centre, age- and sex-matched case–control study was conducted at the Research Department, Clinical Trial Unit, Stavanger University Hospital, from January 1, 2022, to April 1, 2024. Eligible participants were recruited from the Southern Rogaland County population with the support of local general practitioners. Recovered control subjects were primarily recruited through non-familial acquaintances of the patient group or contacts of hospital staff. Time since infection was not a matching criterion but was comparable between groups (*p* = 0.54).

Participants aged 16–80 years with a confirmed SARS-CoV-2 infection (verified via PCR, immunoassay self-testing, or healthcare-provided testing) in line with national guidelines at the time were eligible. Cases had to meet the NICE criteria for LC (symptoms persisting > 12 weeks and not explained by an alternative diagnosis), while recovered controls were defined as individuals with a history of SARS-CoV-2 infection who via clinical interview reported a complete return to baseline health with no lingering symptoms at the time of inclusion.

Exclusion criteria for both LC patients and controls included known autoimmune or chronic inflammatory diseases, cancer, anaemia (haemoglobin < 10 g/dL), hypothyroidism, untreated comorbid conditions affecting fatigue, or inability to provide informed consent or comply with the study protocol. Patients on systemic corticosteroids or other immunomodulatory drugs at the time of the study were also excluded.

Potential candidates were provided with detailed study information and screened for eligibility. All participants who met inclusion criteria provided written informed consent before undergoing clinical examination by a study physician. During this single study visit, clinical and demographic data were recorded, and blood samples collected.

Of the 112 subjects recruited, 52 subjects had LC, while 60 subjects had recovered without sequela. Four subjects in the LC group were excluded: one due to a condition potentially related to vaccine reaction, one with Graves’ disease, one with thyroid cancer, and one with an undiagnosed neurological condition. In the control group, 12 subjects were excluded due to inadequate age and sex matching with cases, including two subjects with anemia and hypothyroidism. After this exclusion process, the final study sample consisted of 48 participants with LC and 48 age- and sex-matched controls, totalling 96 participants, Table 1.

**Table 1:** Selected demographic data of patients with long COVID (n = 48) and recovered controls (n = 48).

	All patients (n = 96)	Long COVID (n = 48)	Controls (n = 48)	*p*-value (LC vs. Ctr)
Age, years (mean, 95%CI)	46.7 (44.2–49.2)	46.9 (43.4–50.3)	46.6 (46.6–42.9)	0.43
Sex, female/male, n (%)	82 (85.4)/14 (14.6)	41 (84.4)/7 (14.6)	41 (84.4)/7 (14.6)	1.0
Time since COVID-19 diagnosis, weeks (median, range)	69.0 (3–188)	65.5 (17–165)	72.5 (3–188)	0.54
Anosmi n (%)		21 (43.7)		
Cough n (%) n = 46		15 (32.6)		
Cough > 4 weeks n = 40		11 (27.5)		
Dyspnea		31 (64.6)		
Memory impairment		46 (95.8)		
Vertigo		33 (68.8)		
Chest pain		19 (39.6)		

### Laboratory tests

Routine haematological and biochemical analyses, including CRP, were performed at the hospital laboratory. Additionally, blood was drawn into EDTA tubes, cooled on ice and centrifuged at 2800 g for 15 min at 4 °C. Plasma was aliquoted and stored at − 80 °C until further analysis.

**Fig. 1:**
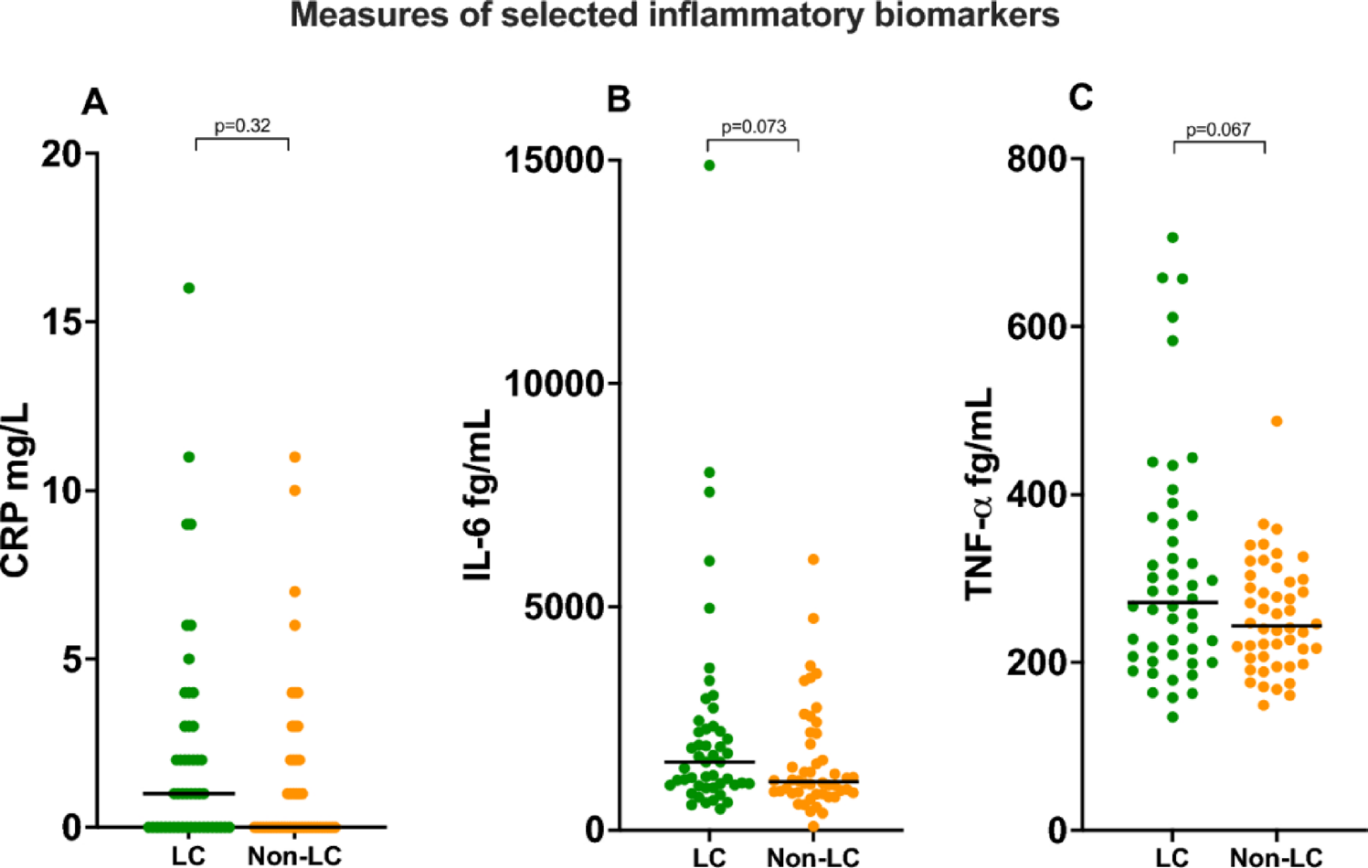
Measures of selected inflammatory biomarkers (median and ranges). CRP measured by hospital’s routine laboratory, IL-6 and TNF-α measured by MSD S-Plex electrochemiluminescence assay. Abbreviations: CRP, C-reactive protein; TNF-α, Tumor necrosis factor-alfa.

The classical proinflammatory cytokines TNF-α, and IL-6 were analysed in duplicate using freshly thawed plasma samples by MSD S-Plex chemiluminescence immunoassay (Meso Scale Diagnostics, Rockville, MD, USA), following the manufacturer’s instructions. Case- and case–control samples were randomly distributed across plates, as were an in-house control sample. For TNF-a, the intra- and inter-assay CV were < 6% (n = 6) and < 7.5% (n = 5), and for IL-6 < 7% (n = 6) and < 12% (n = 5), respectively. According to the manufacturer, the lower limit of quantitation (LLOQ) for TNF-α, and IL-6 are 53 fg/mL and 54 fg/mL, respectively. All measures of TNF-α, and IL-6 were above LLOQ, Fig. 1.

Unfortunately, as the majority of IL-1β analyses yielded concentrations below LLOD, these results had to be omitted.

In addition, ultrasensitive measures of NfL GFAP, TREM2, CRP, TNF-α, IL-1β, and IL6 in plasma were conducted in the Clinical Neurochemistry Laboratory at the University of Gothenburg using the Nucleic Acid Linked Immuno-Sandwich Assay (NULISA™) CNS panel (other analytes not included in this study)^23–25^. For that, plasma samples were thawed on ice and centrifuged at 10,000 g for 10 min and 25uL of the supernatant was added to the analysis plate. After immunocomplex reactions and DNA reporter amplification, the resulting library pool was then sequenced on the Illumina NextSeq 2000 using the standard XLEAP-SBS™ chemistry. The sequencing data were processed following a standard protocol by the manufacturer, which includes intraplate normalisation.

### Data processing and normalization

Sequencing data from NULISAseq was analysed using the NULISAseq algorithm (Alamar Biosciences). Sample (SMI) and target (TMI) barcodes were quantitated, allowing up to two mismatching bases or one indel and one mismatch. Intraplate normalization adjusted each sample’s target counts relative to internal controls, while interplate normalization adjusted counts based on median values from interplate controls. Data was rescaled, with a log2 transformation applied, resulting in NULISA Protein Quantitation (NPQ) units.

### Statistics

Normality of data was tested by Shapiro-Wilks test. Pairwise comparisons were performed using Wilcoxon signed rank test, as most variables showed a non-normal distribution. For the NULISAseq results we applied the Benjamini–Hochberg False Discovery Rate (FDR) to correct for multiple testing (n = 124) to identify significant differences between groups and still maintain a low rate of Type I errors (false positive results). The n = 124 refers to the total number of analytes in the full discovery panel that was run, from which the biomarkers for this study were selected. Both uncorrected and FDR-corrected values are reported, and a p-value of < 0.05 is regarded statistically significant. Confidence intervals for the difference between the groups were also calculated to reflect effect sizes. If the null hypothesis of equality between groups holds, the confidence intervals will include 0, if the null hypothesis does not hold, the confidence interval will quantify the magnitude of the difference. The data were analysed in IBM SPSS Statistics version 26 and R version 4.3.3 (R Core Team, 2024)^26^. Only functions in base R where used in the analyses, including the p.adjust function for FDR.

We selected CRP as the primary biomarker for inflammation due to its established utility in a clinical setting. Given the non-normal distribution of CRP values, we calculated that we had 80% power with a significance level of 0.05 to detect a difference in CRP concentration of at least 2.0 mg/L between cases and control subjects. This effect size was clinically anchored to the observation that the control subjects had CRP concentrations within the normal range (< 5 mg/L), thus we considered this difference as clinically meaningful regarding inflammatory status.

## Results

### Cases and control subjects

Selected clinical characteristics of cases and recovered control subjects are presented in Table 1. Both groups were well-matched for age, sex, and time since SARS-CoV-2 diagnosis, ensuring comparability.

### Inflammatory markers

#### Routine immunoassays (protein concentrations)

CRP levels measured in the hospital’s routine laboratory showed no significant difference between the LC and non-LC groups, with median (range) values of 1.0 (0–16) mg/L vs. 0 (0–11) mg/L, respectively (*p* = 0.32), Fig. 1.

For cytokines measured via MSD electrochemiluminescence assay, levels of IL-6 and TNF-α were marginally higher in the LC group compared to controls, though not reaching statistical significance; IL-6 median (range) 1530 (477–14,900) vs. 1080 (84–6060) fg/mL, * p* = 0.073, and TNF-α 272 (135–706) vs. 244 (149–487) fg/mL, * p* = 0.067, respectively, Fig. 1.

#### NULISAseq (NPQ units)

Using NULISAseq technology to measure biomarker levels, paired uncorrected analyses revealed significantly higher levels of inflammatory markers in the LC group compared to controls: CRP (median: 11.1 vs. 10.2;* p* = 0.035), TNF-α (median: 13.0 vs. 12.8;* p* = 0.007), IL-6 (median: 11.9 vs. 11.3; * p*  =  0.02) and TREM2 (median 12.0 vs. 11.6;* p* = 0.019). Similarly, cerebral biomarkers NfL and GFAP showed no significant differences between groups: NfL (median: 13.7 vs. 13.6;* p* = 0.92) and GFAP (median: 13.8 vs. 13.7; * p* = 0.40).

**Fig. 2:**
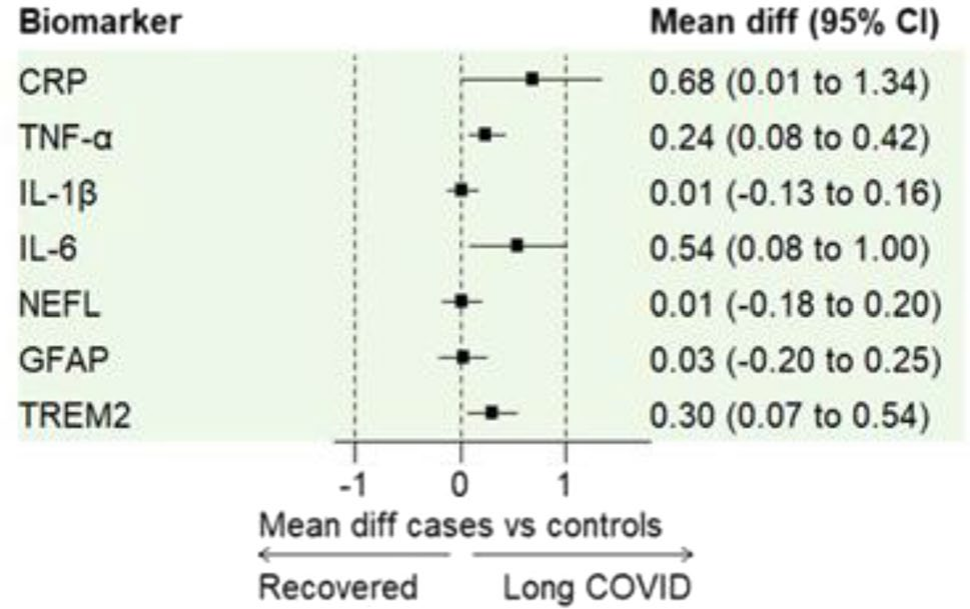
Forest plot illustrating differences in selected inflammatory and neuron damage associated variables between 48 cases with LC and 48 age- and sex- matched control subjects recovered after SARS-CoV-2 infection without long-term effects. Means and the 95% confidence intervals (CI) are indicated. Values > 0 represent higher levels in LC cases. Biomarker concentrations have been analysed as NPQ (NULISA Protein Quantitation) units. Abbreviations: CRP, C-reactive protein; TNF-α, tumor necrosis factor-alfa; IL-1β, interleukin-1 beta; NEFL, neurofilament light; GFAP, glial fibrillary acidic protein.

Estimated mean differences with confidence intervals were as follows: CRP: 0.68, (95% CI 0.01–1.34), TNF-α : 0.245, (95% CI 0.08–0.42), IL-6: 0.544, (95% CI 0.08–1.00), TREM2: 0.30 (95% CI: 0.07–0.54), NfL: 0.01 (95% CI -0.18–0.20) and GFAP: 0.03 (95% CI -0.20–0.25) (Fig. 2). For IL-1β, which showed no significant difference the mean difference was negligible at 0.01; (95% CI -0.13–0.16).

After applying False Discovery Rate (FDR) correction for multiple comparisons, none of the inflammatory biomarkers remained statistically significant between groups: CRP (*p* = 0.12), TNF-α (*p* = 0.05), IL-1β (*p* =  0.88), IL-6 (p = 0.09) and TREM2 (*p* = 0.09) . Similarly, for the cerebral biomarkers the adjusted p-values were as follows: NfL (*p* = 0.96) and GFAP (*p* = 0.64).

### Correlation analysis

We performed Spearman correlation analyses between the inflammatory markers (NfL, GFAP, TREM2, CRP, TNF-α, IL-6) and symptom severity scores in the LC group (n = 48). No significant correlations were observed between any inflammatory biomarker and symptom severity (all * p* > 0.05), indicating that the levels of these markers do not predict clinical symptom burden in this cohort.

## Conclusions

The primary finding of this study is the absence of statistically significant differences in inflammatory or neurological biomarkers between LC patients and recovered controls at a median of 69 weeks post-SARS-CoV-2 infection, when appropriate statistical correction for multiple comparisons is applied. While ultrasensitive NULISA technology detected nominally elevated inflammatory markers in unadjusted analyses, these differences did not survive false discovery rate correction, indicating either no true differences exist or that any differences are too subtle to detect reliably with the current sample size.

Our findings diverge from many earlier studies that reported overt signs of inflammation, immune activation, and organ damage in acute COVID-19^18,19^ , and also in LC^27–29^ . This discrepancy may be attributed to the extended follow-up period in our study, which allowed sufficient time for viral clearance and resolution of acute inflammation. Studies conducted earlier in the disease course, closer to the time of acute infection, may have captured transient inflammatory processes rather than persistent pathology. In contrast, our results align with more recent long-term studies^14–16^, which similarly observed minimal evidence of ongoing inflammation or neurological damage in LC patients over time.

It is possible that one reason why many studies find ongoing inflammation and immune activation in LC is that patient cohorts include individuals with pre-existing autoimmune or chronic inflammatory conditions. In the general population, approximately 5–10% have autoimmune disorders^30^, and these conditions are characterized by many of the same clinical symptoms and inflammatory markers observed in LC. To avoid this potential confounding in our study population, we therefore excluded all patients with known autoimmune or chronic inflammatory diseases. We believe that our patient cohort represents exclusively LC pathophysiology and is not influenced by other conditions that can produce similar inflammatory patterns.

The stability of NfL and GFAP levels between LC patients and controls strongly suggests an absence of significant neuronal damage or astroglial activation. However, NULISA does not provide protein concentrations, but units related to amount of PCR template, so we cannot determine whether these values are elevated compared to reference limits generated outside our study. That said, it seems unlikely that the biomarker levels are abnormally high, as the control group is symptom-free and lacks the findings that characterize the LC group.

### Our findings suggest the following

*No significant neuronal damage*. NfL, a highly sensitive marker for neuronal damage, remained stable across groups. Elevated NfL levels are typically associated with neurodegenerative diseases, traumatic brain injury, or active inflammatory conditions^31^. The lack of elevation in LC patients indicates that ongoing symptoms such as subjective cognitive impairment (“brain fog”) are unlikely to result from structural neuronal damage.

*No evidence of astroglial activation*. GFAP levels were also comparable between groups, suggesting an absence of astrocyte-driven neuroinflammation. Astrocytes are glial cells in the central nervous system (CNS) and play a critical role in maintaining central nervous system (CNS) homeostasis and responding to injury or infection^32^. Their activation is a hallmark of CNS immune responses, accompanied by cytokine production and microglial interaction.

Astrocytes act as both responders to and modulators of immune responses in the CNS. When activated, they interact with microglia, present antigens, and produce cytokines and chemokines. They also respond to viral infections and activate antiviral pathways. As integral players in the innate immune response, astrocytes balance defense and prevention of collateral damage within the CNS environment. The lack of astroglial activation in LC patients suggests an absence of significant immune activation within the CNS.

These observations align with some studies on LC, which show minimal evidence of ongoing CNS damage as assessed by NfL and GFAP levels, and normalization of levels over time^15^ Furthermore, these studies report no differences in neurocognitive function between LC patients and healthy controls, despite a higher symptom burden among the former^16^, but contrasts with some other studies demonstrating neurocognitive impairments^27,28^.

### TREM2 and myeloid activation

TREM2, expressed on microglia and peripheral myeloid cells, has been implicated in SARS-CoV-2 immune responses and microglial activation and is upregulated in acute and severe COVID-19 . However, our finding of normal TREM2 levels in LC, after statistical correction, is notable. While TREM2 is elevated in the acute phase, reflecting active myeloid cell activation, our results suggest that the specific myeloid activation characteristic of the acute infection appears to resolve over time in LC patients. This temporal distinction between acute and chronic phases is important and distinguishes LC from neurodegenerative conditions like Alzheimer’s disease, where sTREM2 remains persistently elevated. This finding contributes novel information to understanding how the immune landscape shifts from the acute to chronic phases of SARS-CoV-2 infection.

### No obvious systemic inflammation

The persistence of cognitive dysfunction (‘brain fog’) in the absence of neurochemical evidence of neuronal injury (normal NfL) or neuroinflammation (normal GFAP, TREM2) suggests that LC symptoms may be functional rather than structural. Metabolic dysfunction at the mitochondrial level could explain these symptoms; if neuronal ATP production is compromised, the brain may enter an “energy conservation mode,” manifesting as cognitive slowing and fatigue^33^. Additionally, symptoms could stem from functional network disconnectivity, where the coordination between brain regions is disrupted despite intact structural connections^34^. Lastly, there is the possibility of dysfunctional central nervous system mechanisms, rather than ongoing tissue damage that drive symptoms such as “brain fog” in the chronic phase. This aligns with the concept of central sensitization, where the brain’s processing of interoceptive signals becomes hypersensitive. Furthermore, psychological factors such as fear-avoidance beliefs and catastrophic interpretations of bodily sensations can perpetuate symptoms by maintaining a state of autonomic hyperarousal and hindering physical reconditioning^35^.

Despite the absence of significant inflammatory differences, the clinical reality of persistent LC symptoms requires explanation. The sickness behavior paradigm provides a compelling theoretical framework. Sickness behavior refers to a coordinated set of behavioral changes—including fatigue, cognitive dysfunction, social withdrawal, and malaise—that develop during infection and are mediated by proinflammatory cytokines, particularly IL-1β and TNF-α. While the magnitude of these changes is insufficient to indicate overt immune activation, they may reflect a chronic, extremely low-level immune activation, that contributes to fatigue, pain, and other sickness symptoms through mechanisms such as pro-inflammatory signaling or epigenetic changes^28,29^.

### Limitations

This study has several limitations. Firstly, the relatively small cohort size may have limited our statistical power to detect subtle biomarker differences. Secondly, our use of a selected biomarker panel means other unmeasured inflammatory pathways could be active. We also acknowledge a potential selection bias, as recruitment through general practitioners may favor patients more actively seeking care.

The study is also limited by its cross-sectional design, which prevents causal inferences, and its reliance on blood-based biomarkers without paired cerebrospinal fluid (CSF) data or functional neuroimaging to confirm central mechanisms. While the NULISA platform provides high sensitivity, it does not report absolute protein concentrations, limiting direct comparisons to reference ranges in healthy populations. Additionally, replicating the findings in an independent cohort would strengthen their validity. Future studies should integrate multimodal assessments-including neuropsychological testing, longitudinal follow-up, and advanced neuroimaging-to track symptom progression and biomarker dynamics over time.

## Conclusion

At a median of 69 weeks post-infection, this study found no significant evidence of peripheral inflammation or neuroinflammation in LC patients compared to recovered controls . While ultrasensitive assays detected nominally elevated inflammatory markers in unadjusted analyses, these findings did not survive rigorous statistical correction, suggesting either no true differences exist or that any differences are below the threshold of reliable detection with current methods and sample size. These results challenge inflammatory hypotheses of LC pathophysiology and highlight the importance using appropriate patient cohorts and rigorous statistical approaches in biomarker research. The findings suggest that LC may involve mechanisms beyond conventional inflammatory pathways, necessitating alternative approaches to understand this complex post-viral syndrome.

## Data Availability

The data that support the findings of this study are not openly available due to reasons of sensitivity and are available from the corresponding author upon reasonable request.
